# From Documents to Digital: Co‐Designing Nutrition Information Videos With Consumers and Dietitians

**DOI:** 10.1111/hex.70576

**Published:** 2026-01-23

**Authors:** Hannah Olufson, Huyen Do, Bridget Noble, Gary Power, Janette Moore, Ruby Ong, Scott Harding, Samantha Robertson, Thilini Gunawardena, Jennifer Ellick, Adrienne Young

**Affiliations:** ^1^ Dietetics & Food Services, Surgical, Treatment & Rehabilitation Service (STARS) Metro North Hospital & Health Service Brisbane Australia; ^2^ STARS Education & Research Alliance STARS, University of Queensland & Metro North Hospital & Health Service Brisbane Australia; ^3^ Nutrition Research Collaborative Royal Brisbane & Women's Hospital Brisbane Australia; ^4^ Centre for Health Services Research University of Queensland Brisbane Australia; ^5^ Consumer Representative Metro North Hospital & Health Service Brisbane Australia; ^6^ Nutrition & Dietetics, Toowoomba Hospital Darling Downs Hospital & Health Service Toowoomba Australia; ^7^ Sunshine Coast Hospital & Health Service Sunshine Coast University Hospital Birtinya Australia; ^8^ Dietetics & Food Services, Royal Brisbane & Women's Hospital Metro North Hospital & Health Service Brisbane Australia

**Keywords:** codesign, dietetics, digital, nutrition, quality improvement

## Abstract

**Introduction:**

Hospital nutrition care is usually supported by offering written, paper‐based information to patients and carers. However, there is a need for evidence‐based online information, such as videos, to support education. We aimed to co‐design nutrition information videos for current and recently discharged hospital patients and share our process for clinicians and researchers undertaking similar initiatives.

**Methods:**

The videos were created by a team of four consumers and six dietitians across Queensland, Australia, who first agreed on the process summarised by a ‘Co‐design Roadmap’. The roadmap guided the development of the video topics, content and creation. The videos were piloted with 15 rehabilitation inpatients at a metropolitan subacute hospital. Feedback was gained on content and perceived knowledge gain via an anonymous questionnaire. The co‐designers completed an anonymous questionnaire to evaluate the process.

**Results:**

Three videos were co‐designed for implementation: ‘Eating When It's Harder to Eat’, ‘Eating for Recovery, Health and Wellness’ and ‘Eating for Stroke Prevention’. Nine patients (60%) who tested the videos reported new learnings, while a further four (27%) felt the videos solidified existing knowledge. Six co‐design team members, excluding the project lead, completed the evaluation questionnaire (66% response rate). All respondents strongly agreed that they felt heard, empowered and equal and that they would participate in similar projects.

**Conclusion:**

Three new nutrition videos were co‐designed, with learning outcomes reported in pilot testing and a positive co‐design experience reported by co‐designers. The ‘Co‐design Roadmap′ that guided this project offers a process for others to use when co‐creating information materials.

**Patient or Public Contribution:**

Previous hospital patients and their caregivers (referred to as ‘consumers′ in this paper) were involved in this co‐design project in various ways. Consumers contributed to the development of the ‘Co‐design Roadmap′, which guided the video co‐creation process. Four consumers were also co‐leads in the video co‐creation process alongside clinicians, with details of their contributions included in this manuscript. Consumers also contributed to the analysis of pilot feedback data and the process of making subsequent changes to improve the video content because of these results. Additionally, consumers had input into the preparation of the manuscript for publication.

## 1 Introduction

Advances in digital technologies have significantly changed how patients access health information, with many turning to online sources of information such as websites, video‐sharing platforms, social media and large language models [1]. Clinical nutrition care is historically supported by mostly non‐digital forms of nutrition information, with patients and carers typically offered paper‐based written nutrition information. However, research increasingly demonstrates the opportunity to move towards digital approaches to information sharing and nutrition care [2, 3]. Suggested benefits of embracing the digitisation of nutrition care include improved patient participation in nutrition care [4], improved nutrition monitoring [5, 6] and possibly enhanced nutrition‐related patient outcomes [7]. Additionally, the need to digitise patient education materials more broadly has recently been highlighted in a scoping review of older patient preferences for education [8]. This review emphasised the need for content to be relevant to the patient group and demonstrate diversity in the images used [7], which are essential to consider in designing digital nutrition resources.

The development of nutrition information materials, like other healthcare resources, policies and research, has traditionally been health practitioner‐ or researcher‐led, with limited input from patients and their caregivers (referred to as ‘consumers′ in this paper) [9]. However, the continued lack of consumer involvement in this process is not acceptable, with the World Health Organization recently stating that it is a human right for end‐users' (in this case, consumers') participation in planning, decision‐making and implementation processes for health across the programme cycle and at all levels of the system [10]. Furthermore, for research organisations and health services, this persistent lack of consumer involvement risks wasted research and resources, such as money and time [9]. In the face of increasing demands on healthcare systems with finite resources, it is time for change.

Research suggests that involving consumers in developing patient education materials (printed, audio‐visual and/or electronic) may result in more relevant and easily understandable resources across different contexts (e.g., hospital and community settings) [11]. One way to achieve this is through the collaborative method of co‐design [12]. The literature cites various definitions and descriptions of co‐design [13]; however, in the context of the International Association of Public Participation, co‐design exists within the ‘Collaborate′ and ‘Empower′ levels of participation [14]. The definition of co‐design adopted for this project is ‘a process where people with professional and lived experience partner as equals to improve health services by listening, learning and making decisions together’ [12]. Co‐design is increasingly used in nutrition interventions and research [13, 15] in line with the recommendations of international health standards [16, 17]. However, notably, many of these studies provide limited details of the steps taken in their co‐design process for application and adoption by others [13].

The increasing digitisation of healthcare supports a move towards digital approaches to nutrition information sharing and the inclusion of consumers in creating these resources to enhance quality. Consumers and dietitians within our local health service together identified a need to co‐design nutrition information videos for current and recently discharged hospital inpatients and their caregivers, with a small hospital foundation grant received to develop these. In line with the recommendations of a recent scoping review [13], this paper aims to describe and evaluate the co‐design process used to create these videos to guide others in undertaking similar initiatives.

## 2 Materials and Methods

**Figure 1:**
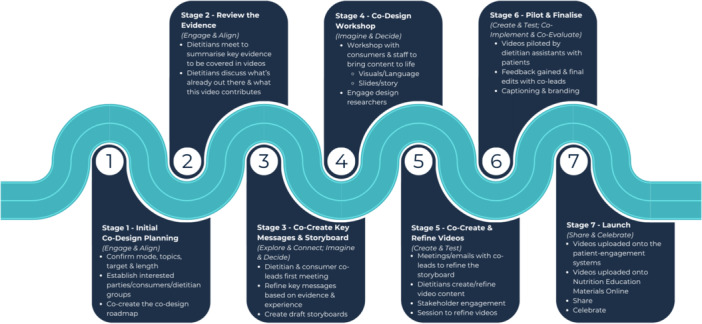
The co‐design roadmap that guided video creation.

This co‐design project was undertaken between January and August 2024. A co‐design approach was chosen for this project to enable people with lived and professional experience to create nutrition education materials together. Specifically, we were guided by the ‘Better Healthcare Together’ Framework (paper under review) developed by consumers and staff within the local health service. This framework suggests a process with six steps: Engage and Align, Explore and Connect, Imagine and Decide, Create and Test, Co‐implement and Co‐evaluate, and Share and Celebrate [12]. Figure 1 provides an overview of how the framework was applied to this project. The protocol for this project was reviewed by the hospital's human research ethics department and approved without requiring review by the ethics committee. The Revised Standards for Quality Improvement Reporting Excellence (SQUIRE 2.0) Checklist [18] and the Guidance for Reporting Involvement of Patients and the Public‐Short Form (GRIPP2‐SF) [19] were used to guide the reporting of this quality activity (see Supplementary Files).

### 2.1 Co‐Design Participants

Overall, there were 16 co‐design participants: seven consumer representatives and nine dietitians (including the project lead/process facilitator). These 16 individuals consulted and contributed to decision‐making throughout the project as part of a broader ‘Co‐design Group′. From this group, four consumers and six dietitians (including the project lead/process facilitator) went on to become ‘Video Development Teams′. Consumers were representatives of the health service and had previously collaborated with the project lead on quality improvement or research projects or were recruited through the local consumer network. All consumers were offered reimbursement according to the health services' payment policy in honour of their contribution to the project; honorariums are to the value of $50–$95 per hour, depending on the activity type. All five dietitian co‐designers were public health service employees recruited by the project lead through existing statewide networks. Aside from the project lead who was funded by a grant from the Royal Brisbane and Women's Hospital Foundation, all dietitians involved provided in‐kind time to this project, supported by their line manager.

The project lead (H.O.) was the process facilitator, ensuring inclusivity and creative participation, maintaining editorial consistency across the videos, and overseeing progress. All ‘Co‐design Group′ members collaborated and provided feedback by making decisions together while following the six principles of co‐design: elevate lived experience, co‐govern the work, be equitable, embrace diversity, promote inclusion and build capacity [13]. Unless otherwise stated, all project decisions were made collaboratively by discussion and consensus among all consumer and clinician members. The project lead facilitated all meetings online with the ‘Co‐design Group′ through Microsoft® Teams between January and August 2024. Monthly steering committee meetings (including five dietitians and two consumers) also occurred online.

#### 2.1.1 Stage 1: Initial Co‐Design Planning (Engage and Align)

Members of the ‘Co‐design Group′ met virtually for 1.5 h to engage, explore and discuss the co‐design process. In this initial meeting, the project lead shared ideas for possible topics for education materials for current or recently discharged hospital inpatients, informed by the findings of local research, service evaluation and consumer engagement activities. The consumers and dietitians decided on three topics: ‘Eating When It′s Harder to Eat’, ‘Eating for Recovery, Health and Wellness’ and ‘Eating for Stroke Prevention’. Following the selection of the topics, the project lead presented further opportunities relevant to the local context to advance nutrition information sharing (i.e., digital systems). The group collaboratively decided the best modality for information provision (videos), target population (creating resources fit for purpose in hospital and at home) and video styles and length. Following this, the locally developed ‘Better Healthcare Together’ Framework [12] was collaboratively transformed into a pragmatic roadmap to guide video development transparently. The ‘Co‐design Roadmap′ (available in Figure 1) provided a visual and practical guide for the team of anticipated tasks, intended outputs and the timeline. It included seven stages, and each stage was followed sequentially.

Given the number of people passionate about the project, consumers decided that the broader ‘Co‐design Group′ would split into smaller ‘Video Development Teams′ with consumer and dietitian co‐leads to progress the creation of each video. The ‘Video Development Teams′ included different dietitians and consumers to leverage specific personal and professional experiences necessary for the different video topics, with the consumer/s of each group having relevant lived experience to the video topic. Additionally, consumers voiced that the dietitians, viewed by them as experts in identifying and synthesising nutrition evidence, should initially summarise and present the evidence for each topic, before working together to prioritise the content and craft the messaging.

#### 2.1.2 Stage 2: Review the Evidence (Engage and Align)

The six dietitians (including the project lead) met virtually in three separate groups (separate 30‐min meeting for each ‘Video Development Team′) to discuss the key evidence for each topic. They worked together offline on documents stored on Microsoft ® OneDrive or via email to identify, review and collate relevant nutrition information and materials regularly used in clinical practice. Key evidence was summarised for each video for discussion and refinement with the consumer co‐leads in a subsequent meeting.

#### 2.1.3 Stage 3: Creating Key Messages and Storyboard (Explore and Connect; Imagine and Decide)

Each ‘Video Development Team′ met virtually with the project lead (separate 1–1.5‐h meeting) to discuss the key evidence compiled by the dietitian co‐leads and translate this into a video storyboard (including audio script and supporting text or visuals). Draft ideas were refined on documents stored on Microsoft ® OneDrive based on nutrition evidence and personal or professional experience to create a draft audio script for each video. The draft audio script was further refined offline by the project lead immediately following each meeting, ensuring these were ready to present back to the ‘Co‐Design Group′.

#### 2.1.4 Stage 4: Co‐Design Workshop (Imagine and Decide)

A 1.5‐h co‐design workshop meeting was held with the broader ‘Co‐Design Group′ to focus on deciding how the content would be brought to life. A summary of the draft audio script for each video and examples of healthcare education videos were shared to inform decision‐making. Video creation software programs recommended by local design researchers were then trialled for usability by the dietitian co‐leads, with consumer feedback sought before selecting (GoAnimate Inc., vyond.com) as the preferred platform. The cost of the Vyond license was funded by the project grant for the project duration.

#### 2.1.5 Stage 5: Co‐Create and Refine (Create and Test)

Each ‘Video Development Team′ then met separately three more times (1.5‐h meetings) to co‐create and refine the video storyboards based on the decisions made in Stage 4. The first of these meetings focused on finalising a draft audio script for each storyboard. Following the meeting, the project lead, one dietitian co‐lead and two student dietitians refined the audio scripts to remove scientific jargon and make the language suitable for people with and without communication difficulties (such as aphasia). At the suggestion of a consumer co‐lead, this process included entering each audio script into ChatGPT (OpenAI, chatgpt.com) with the command: ‘Please make this audio suitable for someone with aphasia’, which provided recommendations used by the project team to guide script editing. Feedback was sought from the respective ‘Video Development Teams′ over email and shared documents on Microsoft OneDrive. Other stakeholders (e.g., members of statewide dietitian networks) were engaged to provide feedback on the audio script before it was finalised.

In the second meeting, ideas for visual content were imagined for each slide to match the audio. Additionally, the type of audio recording (human voice versus artificial intelligence generated text‐to‐speech) was voted on by consumers after examples were provided. The project lead, one dietitian co‐lead and the two student dietitians then worked to transform the storyboard (audio script and proposed visuals) into videos using Vyond. The videos were created and refined using the Patient Education Materials Assessment Tool for Audiovisual Materials (PEMAT‐A/V) [20].

The videos were shared with each ‘Video Development Team′ for final feedback and refinement in the third meeting (1.5 h). This meeting also included sharing and gaining feedback on a local implementation plan with dietitians (not from the hospital where implementation was planned) and consumer stakeholders. Feedback from dietitian stakeholders external to the project was collected through statewide clinical networks (including groups of people from diverse backgrounds) and used to refine the videos. The videos were then shared with the broader ‘Co‐design Group′ in an additional 1.5‐h meeting for final consultation and endorsement.

#### 2.1.6 Stage 6: Pilot and Finalise (Create and Test; Co‐Implement and Co‐Evaluate)

The videos were then uploaded to a video‐sharing platform (Vimeo Inc., vimeo.com; licence available within our health service) and patients were invited to view them and provide their feedback to inform iterative improvements as part of the co‐design process. Patients within a single metropolitan subacute hospital were identified by the ward dietitian as potential end‐users of the videos based on their malnutrition risk score, if they had goals for weight management or were admitted following a stroke. Exclusion criteria included cognitive or vision impairment, receptive aphasia and presence of post‐traumatic amnesia following a brain injury. Five patients were selected to view each video, with the video being shown to them by a dietitian assistant as part of their delivery of nutrition care. Feedback on the video content was collected via a questionnaire developed specifically for this project. The questions were designed for dietitian assistants piloting the videos in their usual clinical workflows to ask patients after showing them the videos. The questionnaire is available in Supporting Information; it was not pilot tested or validated. Dietitian assistants obtained verbal consent from the patients prior to administering the survey and recording their responses in an online version of the questionnaire in Microsoft Forms. Data were analysed using descriptive statistics (counts, %) and any free‐text comments were categorised using inductive content analysis. Feedback from dietitian stakeholders and patients was used to refine the videos, with assistance from the student dietitians and consultation from all members of the ‘Video Development Teams′.

#### 2.1.7 Stage 7: Launch (Share and Celebrate)

A local video premiere event was held at the same metropolitan subacute hospital to launch the videos and celebrate the co‐design process and product. This event coincided with implementing the videos at this site and included both consumer and dietitian co‐leads from the ‘Video Development Teams′ in disseminating the videos, with attendance from internal and external partners. A state‐wide virtual launch was held on Microsoft ® Teams to coincide with uploading videos onto the Nutrition Education Materials Online website (health.qld.gov.au/nutrition) for statewide use. Both consumer and dietitian co‐leads from the ‘Video Development Teams′ contributed to this statewide launch with consumers generously sharing pre‐recorded reflections and insights on the co‐design process.

### 2.2 Evaluation of the Co‐Design Process

A questionnaire was co‐created by members of the ‘Video Development Teams′ to evaluate the co‐design process. At the time of undertaking this project, a co‐design evaluation framework did not exist to evaluate the process. Therefore, the ‘Co‐design Group’® (inclusive of consumers) were presented with a series of questions collated from the ‘Better Healthcare Together’ framework [21] and invited to vote anonymously for their preferred questions via Microsoft Forms. The top 5 questions voted by the group were chosen for the questionnaire, along with one open‐field question to elicit barriers and enablers to the co‐design process. The number of questions included was a pragmatic decision to enhance the acceptability of the questionnaire. The questionnaire is available in Supporting Information; it was not pilot tested or validated.

The questionnaire was disseminated to all members of the Co‐design Group via an anonymous Microsoft ® Forms survey at the conclusion of the co‐design process, with results collated, de‐identified and analysed by a student dietitian. Reasons for non‐participation in questionanaire were not collected. Additionally, the project lead did not complete the questionnaire. Descriptive statistics (counts, %) were used to analyse these data, with inductive content analysis used to categorise any qualitative data from the free‐text comments. Additionally, the consumer co‐authors were invited to reflect on their role in the co‐design process as a contribution to the manuscript. These reflections were guided by the ‘ideal co‐design checklist′ developed by Meloncelli et al. [13].

## 3 Results

Three nutrition information videos were created through this co‐design process, with descriptions and links for each in Table 1. As decided in the first co‐design workshop, videos were 2–4 min long, designed with messaging relevant to consumers in hospitals or at home and their caregivers and included animations. They used easy English language, with audio created through artificial intelligence‐generated text‐to‐speech software in Vyond (GoAnimate Inc.), and animations to represent the audio visually.

**Table 1:** Description and links for each co‐designed nutrition information video.

Video title	Description	Link	Length	PEMAT score
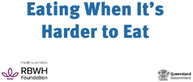	Nutrition tips to support people struggling to eat enough and/or are at risk of malnutrition/or malnourished.	https://vimeo.com/972535203/6ebd2eb1f9	3:02 min	Understandability Score: 92% Actionability Score: 100%
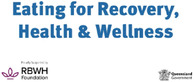	Nutrition tips to support people in the post‐acute recovery or post‐malnutrition phase.	https://vimeo.com/972535106/72e7dccbad	2:39 min	Understandability Score: 92% Actionability Score: 100%
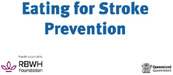	Nutrition tips to help people reduce their risk of having another stroke.	https://vimeo.com/989903273/42d89afb27	2:03 min	Understandability Score: 92% Actionability Score: 100%

Overall, 15 patients provided feedback on the videos during the piloting (*n* = 5 per video). Of the patients who participated in the pilot, 53% (8/15) identified as male, 20% (3/15) were aged 50–59 years, 13% (2/15) were aged 60–69 years and the remaining 67% (10/15) were aged 70+ years. Only 1/15 patients (7%) identified as Aboriginal but not Torres Strait Islander, while the other 14/15 (93%) did not. Overall, regarding the video content, 9/15 patients (60%) reported learning something new from watching one of the videos, and 4/15 (27%) felt it solidified their current knowledge. The remaining 2/15 patients (13%) either did not answer this question or reported not learning anything. Feedback was consistent across the three videos, with most patients reporting that the information was helpful and easy to understand and the video was interesting and of a good length (Table 2).

**Table 2:** Pilot feedback survey results.

	Eating when it's harder to eat			Eating for recovery, health and wellness			Eating for stroke prevention		
	**Response rate (%) (*n*/*N*)**								
Key construct from the survey	Strong Agree /Agree	Neither	Disagree/Strongly Disagree	Strong Agree/Agree	Neither	Disagree/Strongly Disagree	Strong Agree/Agree	Neither	Disagree/Strongly Disagree
Helpful	80 (4/5)	20 (1/5)	0 (0/5)	100 (5/5)	0 (0/5)	0 (0/5)	100 (5/5)	0 (0/5)	0 (0/5)
Easy to understand	100 (5/5)	0 (0/5)	0 (0/5)	100 (5/5)	0 (0/5)	0 (0/5)	100 (5/5)	0 (0/5)	0 (0/5)
Interesting to watch	100 (5/5)	0 (0/5)	0 (0/5)	100 (5/5)	0 (0/5)	0 (0/5)	100 (5/5)	0 (0/5)	0 (0/5)
Good length	100 (5/5)	0 (0/5)	0 (0/5)	100 (5/5)	0 (0/5)	0 (0/5)	100 (5/5)	0 (0/5)	0 (0/5)
Video shown at the appropriate time during hospital admission	100 (5/5)	0 (0/5)	0 (0/5)	80 (4/5)	20 (1/5)	0 (0/5)	100 (5/5)	0 (0/5)	0 (0/5)
Suggested improvement	Nil			Include recipes (20%)			Include information about alcohol intake (40%) Include meal ideas (20%)		
General comments	Had not thought about eating dessert first (40%)			Reiterates prior knowledge about weight management (20%) Liked the plate model (20%)			Learnt about the relationship between food, salt and blood pressure (40%)		

The co‐design process evaluation questionnaire was completed by 6/9 members of the ‘Video Development Teams′ (66% completion rate), with the results displayed in Table 3. All respondents stated ‘Strongly Agree′ to all statements. An open‐ended response revealed enablers and barriers to co‐design participation (see Table 3). The only barrier reported was that scheduling meetings to suit all members was difficult; however, email summaries after the meetings worked well. Enablers cited by participants included ‘I felt that the process had adequate flexibility to allow me to fully participate', I really enjoyed working with the team’ and ‘I really enjoyed the experience and learnt heaps about teamwork, good design process and nutrition’.

**Table 3:** Co‐design evaluation survey results.

Question:	Response rate (%) (*n*/N)				
	Strong Agree	Agree	Neither	Disagree	Strongly Disagree
I feel like this co‐design process has achieved a positive outcome	100 (6/6)	0 (0/6)	0 (0/6)	0 (0/6)	0 (0/6)
I felt heard, respected, engaged, empowered and equal in this co‐design process	100 (6/6)	0 (0/6)	0 (0/6)	0 (0/6)	0 (0/6)
I feel like I had an impact on this co‐design process	100 (6/6)	0 (0/6)	0 (0/6)	0 (0/6)	0 (0/6)
I would choose to be involved in co‐design again in the future	100 (6/6)	0 (0/6)	0 (0/6)	0 (0/6)	0 (0/6)
What could be done to improve/assist your participation in the future co‐design process?	The only trouble was with meeting attendance to suit all members—which is always an issue. I don't know if there's an easier way of scheduling for everyone. In any case, it worked well with email follow‐up. I felt that the process had adequate flexibility to allow me to fully participate. I really enjoyed working with the team. I really enjoyed the experience and learnt heaps about teamwork, good design process and nutrition. The project lead really led the team well through inception to the final product. Nothing. It was excellent. The project team provided good structure, planning and guidance for the co‐design process and consumer involvement. The process, inputs and expected outcomes were very well outlined to both the group as a whole and then to the individual co‐lead. The mood of the meetings was kept light‐hearted, which, I feel, promoted and encouraged the sharing of ideas, experiences and insights with the group. Additionally, the meetings didn't feel rushed; enough time was allowed. The post‐meeting follow‐ups clearly outlined where more input was sought, and sufficient time was provided for follow‐up. Continue with more of the same. It was fun, well organised, and a pleasure to be involved.				

Consumers' reflections provided valuable insights into their experience of the co‐design process (see Box 1). The consumers felt their lived experiences were elevated through the opportunity to share the difficulties they faced regarding their health and nutrition and what helped them overcome these. Notably, most consumers did not have formal involvement in co‐governance responsibilities as reflected in the responses. However, the project lead reported to a project steering committee (comprised of two consumer partners and five dietitians) for 30‐min monthly as a project governance requirement.

Box 1 **Consumer reflections on the co‐design process**

**Elevate lived experience**

- I recall the group started with [a] Nutrition and Mealtime Care Consumer Focus Group where consumers and dietitian brainstormed ideas for future, which included the education videos idea. This then became ‘From Documents to Digital ‐ Co‐designing Nutrition Videos to Improve Patient Care′ project. From a consumer perspective, it was great to be involved in the evolutionary stages as [a] team. We got to brainstorm ideas in both phases. I think hearing everyone's ideas and discussing them as [a] group made everyone's ideas and input important. It seems to set [the] foundation for very collaborative decision‐making.
- I recall eating to prevent secondary stroke was [a] subject I thought in the hospital was important.
- Leverage consumer life experience and expertise, not just [the] lived experience of a health condition.
- The process allowed time for each of us to share our lived experience ‘expertise′, gaining empathy and shared understanding of the difficulties we faced, and still live with.
- Being acknowledged (listened to) created an encouraging environment that prompted a recall of the struggles faced and discussions of what we did, and do, to overcome them, especially in managing adequate nutrition challenges, which fed into great content for improvements and the education videos.

**Co‐govern**

- I don't recall much involvement here. The project progress was presented by [project lead], so I had [an] idea of [the] process. Obviously, a lot of work was being done by [the] professional team to make the project work. Democratising decisions using polls and discussion worked very well, I thought. Respecting everyone's opinions was important. It was great to have discussions where the dietitians could provide expertise and professionally guide the group, and we could share our experience. I thought it was important to learn from the dietitian's experience with patients as well.
- Being a co‐lead didn't include co‐governance responsibilities (I don't think!). The project requirements were communicated well for each step of the project. It made my involvement unpressured. The project [lead] continually updated the team, providing overviews to ensure consistency across the project and was receptive to any suggestions. I think it would be counterproductive for me and the project to be too involved in governance aspects.

**Equity‐centric**

- The process adopted gave everyone a chance to share and have equity in the project. We talked a lot in the main meetings [with the broader Co‐design Group] about our experiences, our thoughts on the videos and ideas for improvement.
- In the smaller video groups [Video Development Team], I felt I was part of [a] team. I think all in the team appreciated [that] they had an important role. There was a free exchange of comments and ideas between consumers and professionals. There were times I appreciated [the] role of [health] professionals and listened and I felt that reciprocated.
- I think no one was wrong, so we could share ideas generously and decide if they worked as a team. This gave everyone equity and enriched experience.

**Diversity**

- Sought feedback from wider stakeholders from culturally diverse backgrounds to enhance the applicability of the video content across diverse groups.
- I am not too sure about diversity statistics. The whole group seemed focused on improving health outcomes, which perhaps reflects a lack of diversity. Maybe the group could have benefited from people who were chronically obese, lower socio‐economic [status], limited diet choices and other comorbidities.
- The meetings and workshops brought together people of diverse backgrounds in terms of age, culture, illness and recovery experience, medical experience, seniority, location, and professional life experience, which enabled all of us to tap into our whole life experiences every day to share, learn and refine our inputs.

**Inclusion**

- Recruited consumer representatives from the local health service.
- Recruited dietitians from statewide networks with professional expertise according to each video topic.
- From very early in the development process, it felt like we were walking in each other's shoes.
- A testament to the coordination, organisation and leadership of the team, willingness and understanding of the need for teamwork amongst all participants—feels like we put our egos aside.

**Building capability**

- A consumer's [with aphasia] suggestion was to use artificial intelligence to simplify the language and make it appropriate for those with aphasia.
- Creation of videos despite no prior experience in video creation/graphics design.
- One very enjoyable part of the experience was to learn a lot: consumer experiences, leadership skills, diet, working in teams and enjoying synergy, learning to listen and having good discussions, helping people, making friends, sharing ideas, giving ideas, but letting the team find answers.
- Once the animation tool and artificial intelligence‐generated voice were decided upon, ideas, suggestions and refinements in the video and messaging could be, and were, implemented quickly and effectively. It was [a] fun process in learning and trialling how we could incorporate a wealth of nutritional education and rehabilitation experience and make it relevant to a patient's possible circumstances in a short animation.
- We all had something to add, consider and reevaluate, in an environment comfortable to do so—we listened to each other.
- It is hoped [that] the animation software becomes a standard which [health service] funds the licences, for updating the videos and creating new material for other topics.

While our co‐design team comprised more healthcare professionals compared to consumers (and therefore did not meet the description for being equity‐centric), the consumers still reported having equal and important input in the project (see Box 1). The project lead recruited a range of consumers, dietitians and other stakeholders, bringing together people of various backgrounds such as age, communication needs, cultural background, illness and recovery experience, professional experience, seniority, and health service location. The project lead also provided a platform for inclusive practice through welcoming and valuing each team member, which enabled participation and achievement of project goals. By recognising that everyone has something to share and teach, this allowed all involved to tap into their personal and professional experiences and promoted the ability to learn, unlearn and re‐learn together, which built capacity within the team. An example of a learning from this project was the suggestion from one consumer to simplify the language (audio and text) to make it more appropriate for those with aphasia through using artificial intelligence.

## 4 Discussion

Through a co‐design process, we have developed three new nutrition information videos in which consumers and dietitians translated nutrition evidence into simple, engaging messages. These videos are now published online and are freely available for use; the links are provided in Table 1. Initial pilot testing suggests that the messaging in the videos is acceptable, with most patients who viewed the videos reporting positive learning outcomes. These results support the findings of Nilsen et al. regarding the benefit of involving consumers in developing relevant and easily understandable patient education materials [11]. Furthermore, these videos are a potentially valuable online resource for dietitians to share with patients, with reassurance that the messaging is likely understandable and relevant as it was developed in partnership with end‐users. These materials may help to address an identified need for online evidence‐based nutrition information to direct patients to, particularly in resource‐constrained settings or where patients and caregivers are seeking online information [22]. It is important, however, to note that some patient groups may not access digital health education materials (e.g., older adults and lower health literacy [23]), necessitating various forms of education to promote person‐centred nutrition care.

Through this co‐design process, consumers and dietitians worked together as partners to decide the project direction, plan the video development method, design the videos, launch the videos and disseminate the work (including as co‐authors on this paper). As part of this partnership, we developed the ‘Co‐design Roadmap′ in which we invite other healthcare professionals, consumers or researchers to use when co‐designing healthcare resources with end‐users. The success of this partnership was reflected in our co‐design evaluation, where participants agreed that a positive outcome had been achieved and reflected positive interpersonal experiences and interactions such as feelings of being heard, respected, engaged, empowered and equal. These emotions are often linked to feelings of being valued and treated well in relationships or group settings and are therefore essential for building trust, cooperation and positive relationships, which underpin meaningful co‐design [23].

Barriers to healthcare co‐design from the literature include challenges of the perceived hierarchy between health professionals and consumers [11]. Namely, while health professionals intend to work in partnership with consumers, they may view themselves as authorities, which creates an inequality in power sharing [11]. This may cause consumers to feel that their input and influence are minimised and may not find it meaningful to participate in co‐design. Additionally, a lack of consumer engagement can result in a mismatch between the wants and needs of the consumers versus the outputs produced [13]. We attempted to address these barriers by involving consumers from the outset and across the different stages of the project. We also worked towards fulfilling the ‘ideal co‐design checklist criteria′ through elevating the experiences of our consumers and building diversity, inclusivity and capacity within the team. We did not meet the criteria for being equity‐centric as the number of consumers involved in the project team was less than the number of dietitians; however, this provides an opportunity for improvement in future projects. Despite one consumer sharing feedback about the logistical challenges of scheduling meetings to suit every member, all consumers attended most meetings and were remunerated for their time, thereby elevating their expertise and contributions to the project.

There are limitations to this project which are important to consider. The published videos are only available in English due to the additional cost of translating them into other languages; however, there remains the ability to translate them into other languages with further funding. We only tested the videos in a very small number of selected patients in one metropolitan subacute hospital, limiting any conclusions about the effectiveness of the videos to change learning, behavioural or nutritional outcomes. This was outside the scope of this evaluation and is an opportunity for future research with larger, more representative samples and the use of validated measures. Additionally, the percentages provided in Section Results, 3 are biased due to small denominators and should be interpreted in the context of the denominators as indicated alongside estimates in text and tables. Whilst the co‐design process included seeking feedback from stakeholders from culturally diverse backgrounds to enhance the content of the videos (e.g., incorporating more plant‐based high protein food options), another limitation of the small sample size is the lack of cultural diversity within the participants of the pilot testing; this should be a focus of future research. Artificial intelligence was used to support video development; however, its impact on the final videos was not specifically evaluated in this study. There are also limitations with the evaluation of the co‐design process. As identified by the consumers themselves, there was a lack of cultural diversity within the co‐design teams, as well as a high health literacy amongst consumers. In the absence of an existing co‐design evaluation framework at the time of the study, we used locally developed evaluation measures co‐designed with the co‐design team. Whilst using this questionnaire may have enhanced the relevance of these measures, the validity and reliability of the findings are not known. Further, we also acknowledge that there is a risk of response bias given the relationships built through the co‐design process and potential hesitancy to report negative experiences, despite the anonymous nature of the evaluation and use of an external person to deploy and analyse the questionnaire. Since the time of our evaluation, a co‐design evaluation framework [9] has been published, which may promote evaluation rigour, validity and the ability to compare data across co‐design projects.

## 5 Conclusion

Nutrition information videos were created through a collaborative, co‐designed approach, with positive outcomes and experiences reported from the videos and the process taken to create them. Future research should be undertaken to determine the effectiveness of co‐designed nutrition information on learning, behavioural or nutritional outcomes with larger, more representative samples and the use of validated measures. Consumers involved in this project felt their contribution was meaningful and that their voices were heard, which produced a positive impact on the project. We encourage the sharing and use of the ‘Co‐Design Roadmap′ employed in this project to guide the co‐design of other healthcare information resources, supporting the involvement of end‐users from the outset.

## Author Contributions

**Hannah Olufson:** conceptualisation, methodology, formal analysis, resources, writing – original draft, writing – review and editing, visualisation, data curation, investigation, project administration, funding acquisition. **Huyen Do:** methodology, formal analysis, writing – original draft, writing – review and editing, visualisation, project administration. **Bridget Noble:** conceptualisation, methodology, formal analysis, writing – original draft (consumer reflections). **Gary Power:** conceptualisation, methodology, formal analysis, writing – original draft (consumer reflections), funding acquisition. **Janette Moore:** conceptualisation, methodology, formal analysis, writing – original draft (consumer reflections). **Ruby Ong:** methodology, formal analysis, writing – original draft, writing – review and editing. **Scott Harding:** conceptualisation, methodology, formal analysis, writing – original draft (consumer reflections), writing – review and editing. **Samantha Robertson:** conceptualisation, methodology, formal analysis, writing – original draft, writing – review and editing. **Thilini Gunawardena:** methodology, formal analysis, writing – original draft, writing – review and editing. **Jennifer Ellick:** conceptualisation, methodology, formal analysis, resources, writing – review and editing, supervision, funding acquisition. **Adrienne Young:** conceptualisation, methodology, formal analysis, writing – review and editing, visualisation, supervision, funding acquisition.

## Ethics Statement

The protocol for this project was reviewed by the hospital's human research ethics department and approved without requiring review by the ethics committee.

## Conflicts of Interest

The authors declare no conflicts of interest.

## Supporting information

Supplementary Files_final.

## Data Availability

The data that support the findings of this study are available on request from the corresponding author. The data are not publicly available due to privacy or ethical restrictions.
