# Supplementary material for: From Documents to Digital: Co‐Designing Nutrition Information Videos With Consumers and Dietitians
Source: Health Expect. 2026 Jan 23;29(1):e70576. doi: 10.1111/hex.70576 (PMC12828781; doi:10.1111/hex.70576)
Supplement: Supplementary file 1 — Supplementary Files_final. [file HEX-29-e70576-s001.docx]

**Supplementary Files**

**Supplementary File 1: GRIPP2 Checklist**

From: [GRIPP2 reporting checklists: tools to improve reporting of patient and public involvement in research](https://researchinvolvement.biomedcentral.com/articles/10.1186/s40900-017-0062-2)

| **Section and topic** | **Item** | **Reported on page No** |
| --- | --- | --- |
| 1: Aim | Report the aim of PPI in the study | 1-3 |
| 2: Methods | Provide a clear description of the methods used for PPI in the study | 3-8  Box 1 |
| 3: Study results | Outcomes—Report the results of PPI in the study, including both positive and negative outcomes | 8-10, Table 3 and  Box 1 |
| 4: Discussion and conclusions | Outcomes—Comment on the extent to which PPI influenced the study overall. Describe positive and negative effects | 10-12  Box 1 |
| 5: Reflections/critical perspective | Comment critically on the study, reflecting on the things that went well and those that did not, so others can learn from this experience | 10-12, Table 3 and  Box 1 |

1. *PPI* patient and public involvement

**Supplementary File 2: SQUIRE Checklist**

# Reporting checklist for quality improvement in health care.

Based on the SQUIRE guidelines.

## Instructions to authors

Complete this checklist by entering the page numbers from your manuscript where readers will find each of the items listed below.

Your article may not currently address all the items on the checklist. Please modify your text to include the missing information. If you are certain that an item does not apply, please write "n/a" and provide a short explanation.

Upload your completed checklist as an extra file when you submit to a journal.

In your methods section, say that you used the SQUIREreporting guidelines, and cite them as:

Ogrinc G, Davies L, Goodman D, Batalden P, Davidoff F, Stevens D. SQUIRE 2.0 (Standards for QUality Improvement Reporting Excellence): revised publication guidelines from a detailed consensus process

|  |  | Reporting Item | Page Number |
| --- | --- | --- | --- |
| **Title** |  |  |  |
|  | [#1](https://www.goodreports.org/reporting-checklists/squire/info/#1) | Indicate that the manuscript concerns an initiative to improve healthcare (broadly defined to include the quality, safety, effectiveness, patient centeredness, timeliness, cost, efficiency, and equity of healthcare) | 1 |
| **Abstract** |  |  |  |
|  | [#02a](https://www.goodreports.org/reporting-checklists/squire/info/#02a) | Provide adequate information to aid in searching and indexing | 1 |
|  | [#02b](https://www.goodreports.org/reporting-checklists/squire/info/#02b) | Summarize all key information from various sections of the text using the abstract format of the intended publication or a structured summary such as: background, local problem, methods, interventions, results, conclusions |  |
| **Introduction** |  |  |  |
| Problem description | [#3](https://www.goodreports.org/reporting-checklists/squire/info/#3) | Nature and significance of the local problem | 2-3 |
| Available knowledge | [#4](https://www.goodreports.org/reporting-checklists/squire/info/#4) | Summary of what is currently known about the problem, including relevant previous studies | 2-3 |
| Rationale | [#5](https://www.goodreports.org/reporting-checklists/squire/info/#5) | Informal or formal frameworks, models, concepts, and / or theories used to explain the problem, any reasons or assumptions that were used to develop the intervention(s), and reasons why the intervention(s) was expected to work | 2-3 |
| Specific aims | [#6](https://www.goodreports.org/reporting-checklists/squire/info/#6) | Purpose of the project and of this report | 2-3 |
| **Methods** |  |  |  |
| Context | [#7](https://www.goodreports.org/reporting-checklists/squire/info/#7) | Contextual elements considered important at the outset of introducing the intervention(s) | 3 |
| Intervention(s) | [#08a](https://www.goodreports.org/reporting-checklists/squire/info/#08a) | Description of the intervention(s) in sufficient detail that others could reproduce it | 3-8  Box 1 |
| Intervention(s) | [#08b](https://www.goodreports.org/reporting-checklists/squire/info/#08b) | Specifics of the team involved in the work | 3-8  Box 1 |
| Study of the Intervention(s) | [#09a](https://www.goodreports.org/reporting-checklists/squire/info/#09a) | Approach chosen for assessing the impact of the intervention(s) | 3-8 |
| Study of the Intervention(s) | [#09b](https://www.goodreports.org/reporting-checklists/squire/info/#09b) | Approach used to establish whether the observed outcomes were due to the intervention(s) | 3-8 |
| Measures | [#10a](https://www.goodreports.org/reporting-checklists/squire/info/#10a) | Measures chosen for studying processes and outcomes of the intervention(s), including rationale for choosing them, their operational definitions, and their validity and reliability | 3-8 and supplementary files |
| Measures | [#10b](https://www.goodreports.org/reporting-checklists/squire/info/#10b) | Description of the approach to the ongoing assessment of contextual elements that contributed to the success, failure, efficiency, and cost | 3-8 and supplementary files |
| Measures | [#10c](https://www.goodreports.org/reporting-checklists/squire/info/#10c) | Methods employed for assessing completeness and accuracy of data | 3-8 |
| Analysis | [#11a](https://www.goodreports.org/reporting-checklists/squire/info/#11a) | Qualitative and quantitative methods used to draw inferences from the data | 3-8 |
| Analysis | [#11b](https://www.goodreports.org/reporting-checklists/squire/info/#11b) | Methods for understanding variation within the data, including the effects of time as a variable | 3-8 |
| Ethical considerations | [#12](https://www.goodreports.org/reporting-checklists/squire/info/#12) | Ethical aspects of implementing and studying the intervention(s) and how they were addressed, including, but not limited to, formal ethics review and potential conflict(s) of interest | 3-8 |
| **Results** |  |  |  |
|  | [#13a](https://www.goodreports.org/reporting-checklists/squire/info/#13a) | Initial steps of the intervention(s) and their evolution over time (e.g., time-line diagram, flow chart, or table), including modifications made to the intervention during the project | 8-10  Tables 2 & 3 |
|  | [#13b](https://www.goodreports.org/reporting-checklists/squire/info/#13b) | Details of the process measures and outcome | 8-10  Tables 2, 3, 4 |
|  | [#13c](https://www.goodreports.org/reporting-checklists/squire/info/#13c) | Contextual elements that interacted with the intervention(s) | 8-10  Box 1 |
|  | [#13d](https://www.goodreports.org/reporting-checklists/squire/info/#13d) | Observed associations between outcomes, interventions, and relevant contextual elements | N/A |
|  | [#13e](https://www.goodreports.org/reporting-checklists/squire/info/#13e) | Unintended consequences such as unexpected benefits, problems, failures, or costs associated with the intervention(s). | 8-10 |
|  | [#13f](https://www.goodreports.org/reporting-checklists/squire/info/#13f) | Details about missing data | 8-10 |
| **Discussion** |  |  |  |
| Summary | [#14a](https://www.goodreports.org/reporting-checklists/squire/info/#14a) | Key findings, including relevance to the rationale and specific aims | 10-12 |
| Summary | [#14b](https://www.goodreports.org/reporting-checklists/squire/info/#14b) | Particular strengths of the project | 10-12 |
| Interpretation | [#15a](https://www.goodreports.org/reporting-checklists/squire/info/#15a) | Nature of the association between the intervention(s) and the outcomes | 10-12 |
| Interpretation | [#15b](https://www.goodreports.org/reporting-checklists/squire/info/#15b) | Comparison of results with findings from other publications | 10-12 |
| Interpretation | [#15c](https://www.goodreports.org/reporting-checklists/squire/info/#15c) | Impact of the project on people and systems | 10-12 |
| Interpretation | [#15d](https://www.goodreports.org/reporting-checklists/squire/info/#15d) | Reasons for any differences between observed and anticipated outcomes, including the influence of context | 10-12 |
| Interpretation | [#15e](https://www.goodreports.org/reporting-checklists/squire/info/#15e) | Costs and strategic trade-offs, including opportunity costs | N/A |
| Limitations | [#16a](https://www.goodreports.org/reporting-checklists/squire/info/#16a) | Limits to the generalizability of the work | 11-12 |
| Limitations | [#16b](https://www.goodreports.org/reporting-checklists/squire/info/#16b) | Factors that might have limited internal validity such as confounding, bias, or imprecision in the design, methods, measurement, or analysis | 11-12 |
| Limitations | [#16c](https://www.goodreports.org/reporting-checklists/squire/info/#16c) | Efforts made to minimize and adjust for limitations | 11-12 |
| Conclusion | [#17a](https://www.goodreports.org/reporting-checklists/squire/info/#17a) | Usefulness of the work | 11-12 |
| Conclusion | [#17b](https://www.goodreports.org/reporting-checklists/squire/info/#17b) | Sustainability | 11-12 |
| Conclusion | [#17c](https://www.goodreports.org/reporting-checklists/squire/info/#17c) | Potential for spread to other contexts | 11-12 |
| Conclusion | [#17d](https://www.goodreports.org/reporting-checklists/squire/info/#17d) | Implications for practice and for further study in the field | 12 |
| Conclusion | [#17e](https://www.goodreports.org/reporting-checklists/squire/info/#17e) | Suggested next steps | 12 |
| **Other information** |  |  |  |
| Funding | [#18](https://www.goodreports.org/reporting-checklists/squire/info/#18) | Sources of funding that supported this work. Role, if any, of the funding organization in the design, implementation, interpretation, and reporting | Title Page |

None The SQUIRE 2.0 checklist is distributed under the terms of the Creative Commons Attribution License CC BY-NC 4.0. This checklist can be completed online using <https://www.goodreports.org/>, a tool made by the [EQUATOR Network](https://www.equator-network.org) in collaboration with [Penelope.ai](https://www.penelope.ai)

**Supplementary File 3: Video piloting feedback survey for staff to complete with patients**

*Part 1: Questions for staff to ask patients after showing them the video*

**Question 1**: Which video are you collecting feedback for?
Eating for Stroke Prevention
Eating When It’s Harder to Eat
Eating for Health, Recovery and Wellness

**Question 2**: The information in the video was helpful
Strongly Agree
Agree
Neutral
Disagree
Strongly Disagree

**Question 3**: The information in the video was easy to understand  
Strongly Agree
Agree
Neutral
Disagree
Strongly Disagree

**Question 4**: The video is interesting to watch (e.g. pictures, use of colour, formatting)
Strongly Agree
Agree
Neutral
Disagree
Strongly Disagree

**Question 5**: The video is a good length
Strongly Agree
Agree
Neutral
Disagree
Strongly Disagree

**Question 6**: The words are large enough to read
Strongly Agree
Agree
Neutral
Disagree
Strongly Disagree

**Question 7**: The video was shown at an appropriate time during my hospital stay
Strongly Agree
Agree
Neutral
Disagree
Strongly Disagree

**Question 8:** What's one thing you learnt from the video?
Free text response

**Question 9:** Is there any information you would like to see included OR removed in this video
Free text response

**Question 10:** Do you have any other comments? *please record any other comments the patient has here*
Free text response

*Part 2: Demographic data*

**Question 11:** What is your age?
16-24
25-29
30-39
40-39
50-59
60-69
70+
Prefer not to state

**Question 12**: What gender do you identify as?
Male
Female
Intersex
Prefer not to state

**Question 13:** Are you of Aboriginal or Torres Strait Islander origin?
Yes, Aboriginal
Yes, Torres Strait Islander
Yes, both Aboriginal and Torres Strait Islander
Neither
Prefer not to state

**Supplementary File 4: Co-design process evaluation questionnaire**

**Question 1:** I feel like this co-design process has achieved a positive outcome
Strongly Agree
Agree
Neutral
Disagree
Strongly Disagree

**Question 2:** I felt herd, respected, engaged, empowered and equal in this co-design process
Strongly Agree
Agree
Neutral
Disagree
Strongly Disagree

**Question 3:** I feel like I had an impact in this co-design process
Strongly Agree
Agree
Neutral
Disagree
Strongly Disagree

**Question 4:** I would choose to be involved in co-design again in the future
Strongly Agree
Agree
Neutral
Disagree
Strongly Disagree

**Question 5:** What could be done to improve/assist your participation in future co-design processes? Free text response
